# A Vesicocutaneous Fistula Treated With Urinary Diversion, Negative Pressure Wound Therapy, and Time

**DOI:** 10.7759/cureus.65313

**Published:** 2024-07-24

**Authors:** Adham Al-Hajj, Wassim Hamze, Georgio El Koubayati, Fady Haddad, Antoine Noujeim

**Affiliations:** 1 Department of Urology, Faculty of Medical Sciences, Lebanese University, Beirut, LBN; 2 Department of Surgery, Division of Urology, Lebanese Hospital Geitaoui, Beirut, LBN; 3 Department of Internal Medicine, Division of Clinical Immunology, Faculty of Medical Sciences, Lebanese University, Beirut, LBN; 4 Department of Internal Medicine, Division of Clinical Immunology, Lebanese Hospital Geitaoui, Beirut, LBN

**Keywords:** vesicocutaneous fistula, negative pressure wound therapy, complicated urinary tract infection, urine leak, trauma, fistula

## Abstract

A 38-year-old motor vehicle accident victim presented for acute urinary retention due to a clogged Foley catheter, which was inserted two weeks prior during surgery for pelvic and spine fixation and extra-peritoneal bladder rupture. Imaging studies revealed persistent bladder leaks despite primary and, later, secondary surgical repair. A combination of novel non-surgical techniques, that is, urinary diversion, negative pressure dressings, and waiting proved beneficial in our case, and led ultimately to complete clinical and radiological resolution of the fistula.

## Introduction

A vesicocutaneous fistula (VCF) is a rare entity where an abnormal tract connects the bladder and the skin leading to a continuous urinary leak. It usually arises following non-healing bladder injuries caused by pelvic trauma, prior pelvic surgery, pelvic radiation therapy, infection, congenital anomalies and other causes that have been reported [1]. VCFs are complex by nature and their treatment is challenging. Given the preexisting morbid setting in which they occur, simple surgical repair is often difficult and unsuccessful [2]. Additionally, a clear management directive is absent, probably due to the heterogeneity of cases. Hereby, we report the case of a 38-year-old male, a victim of a motor vehicle accident, who developed a VCF between the bladder and the suprapubic region after a non-healing bladder rupture.

## Case presentation

**Figure 1:**
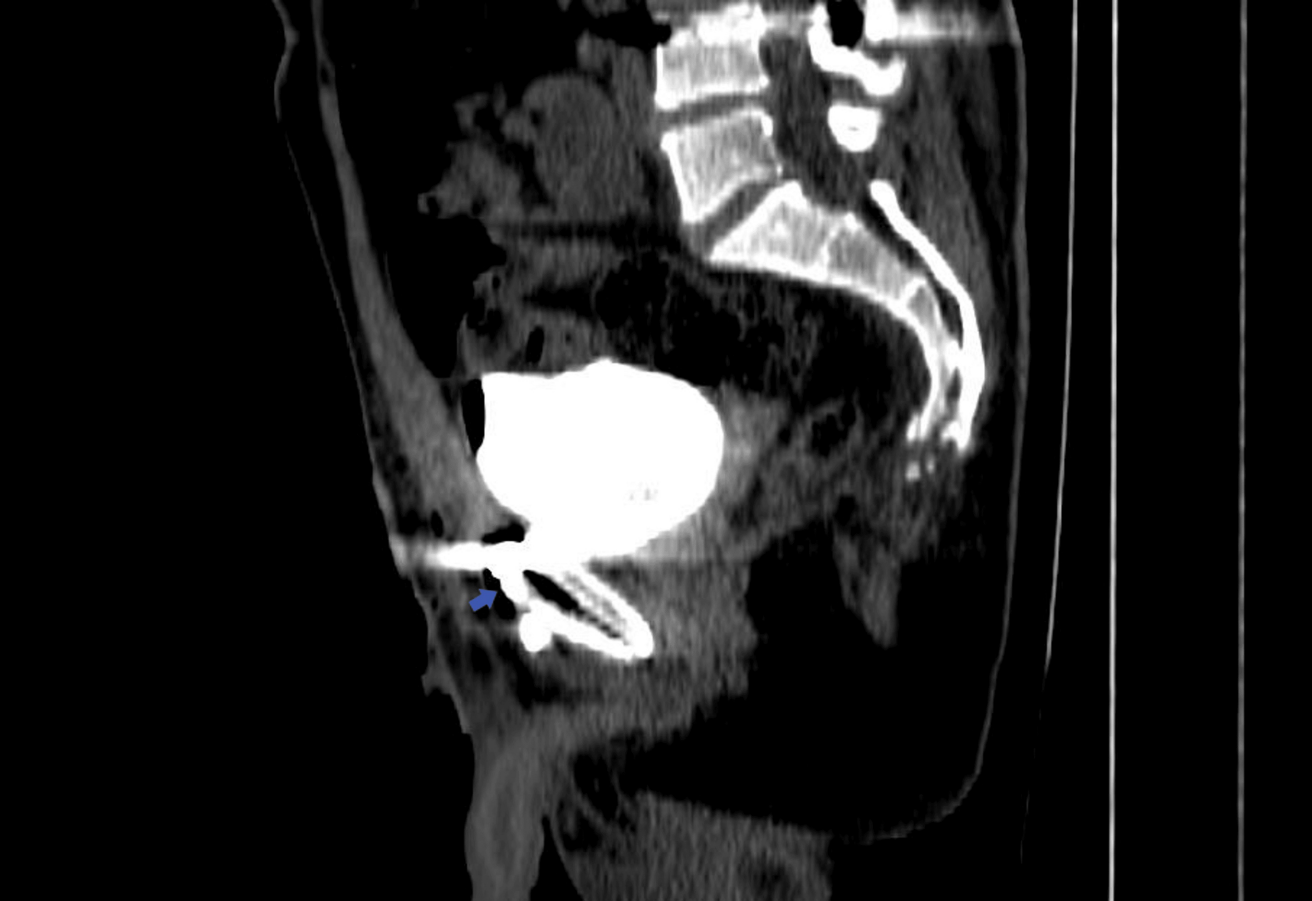
CT scan of the abdomen and pelvis, delayed phase, showing anterior bladder rupture with contrast extravasation (arrow) into retropubic space

A 38-year-old Lebanese male patient presented to the emergency department of the Lebanese Hospital Geitaoui for severe abdominal pain and suprapubic tenderness that started 12 hours prior. The patient had an indwelling Foley catheter. Upon presentation, the patient had normal vital signs but looked in distress with gross hematuria visible in the urine bag. His abdomen was soft with localized suprapubic rigidity and tenderness. A Pfannenstiel incision with surgical staples and a vertical lumbar incision from previous surgery were seen. Urinary retention was suspected due to a clogged catheter, which was replaced in the emergency department. The patient was immediately relieved and 600 ml of dark bloody urine was immediately drained. His history goes back to two weeks prior when he was the victim of a high-speed motor vehicle accident, which resulted in multiple pelvic and lumbar vertebral fractures as well as extra-peritoneal bladder rupture. Fixation of the pelvis and instrumentation of the spine along with repair of the extra-peritoneal bladder rupture was done at the time in another institution. A Foley catheter was inserted postoperatively and the patient was discharged. Blood tests upon presentation were within normal limits and urine analysis showed numerous red blood cells and white blood cells. A CT scan of the abdomen and pelvis with IV contrast was ordered in the emergency department (Figure 1), an extra-peritoneal anterior bladder wall rupture was visualized with evidence of contrast leak into the retro-pubic fat on the delayed phase, a metallic plate and screws over the pubic symphysis, a fracture of the anterior third of the right iliac wing and posterior spondylodesis of L2 to L5 with good alignment of vertebral bodies. The patient was admitted to the general medicine ward for extra-peritoneal bladder rupture. He was started on third-generation cephalosporins awaiting urine culture results.

**Figure 2:**
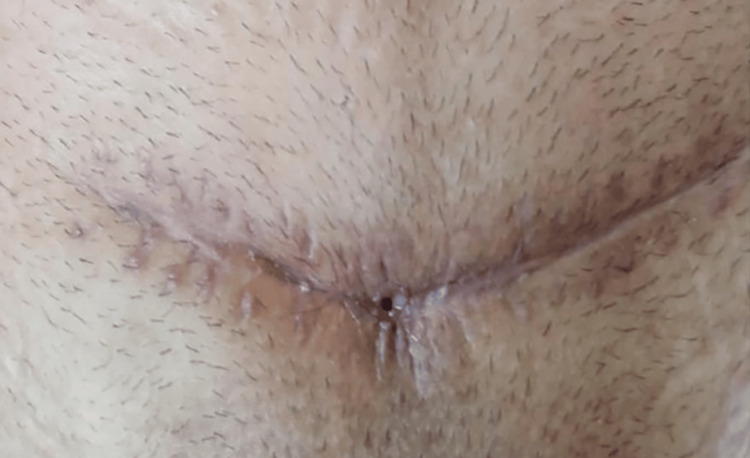
Pfannenstiel incision wound, with the VCF opening visible in the middle VCF: vesicocutaneous fistula

Ten days after admission, urine leakage from the suprapubic wound began despite the presence of the Foley catheter. A 5 mm opening of the fistula can be seen in the middle of the wound (Figure 2).

**Figure 3:**
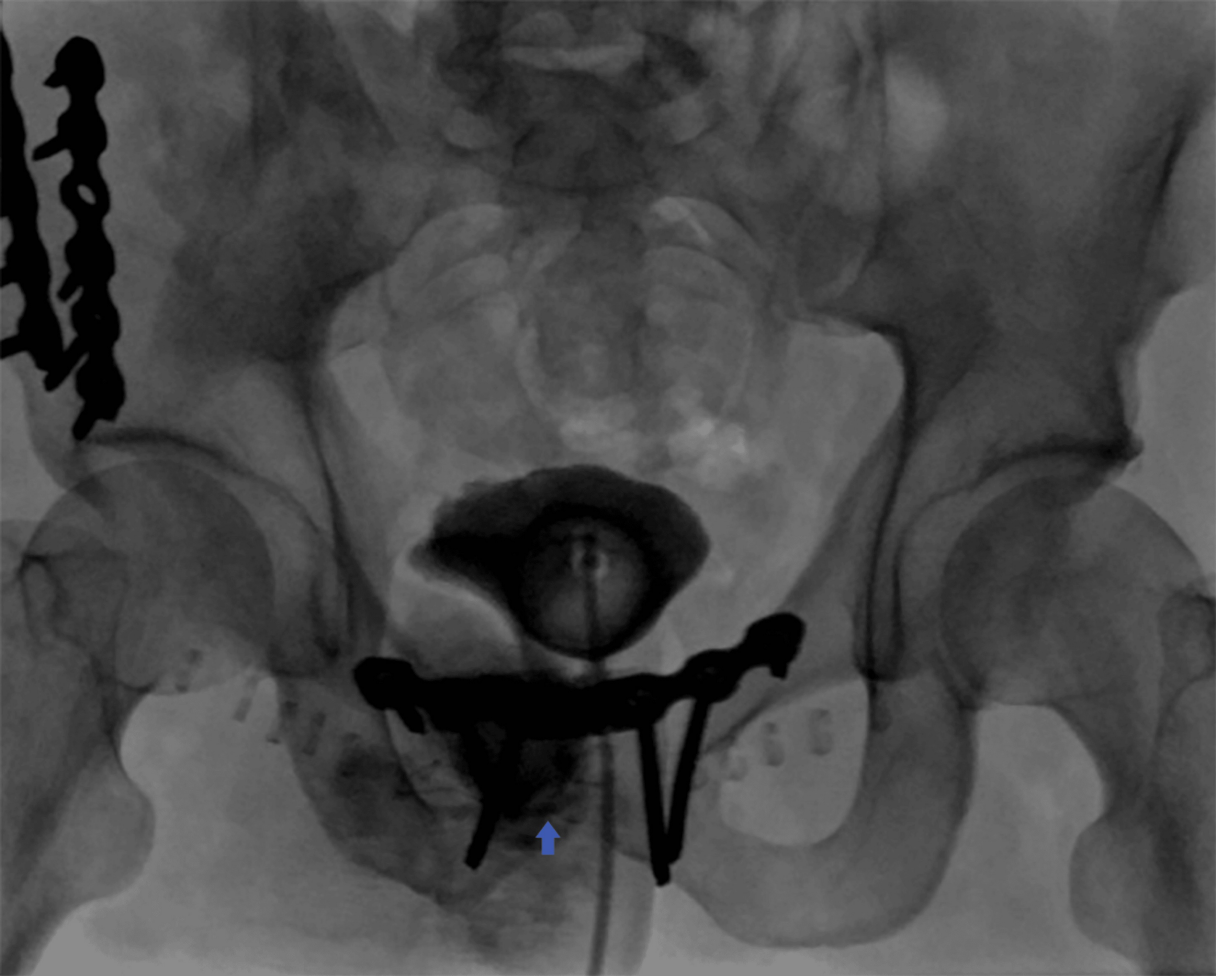
Retrograde cystography showing contrast extravasation (arrow) following bladder inflation, anterior view

**Figure 4:**
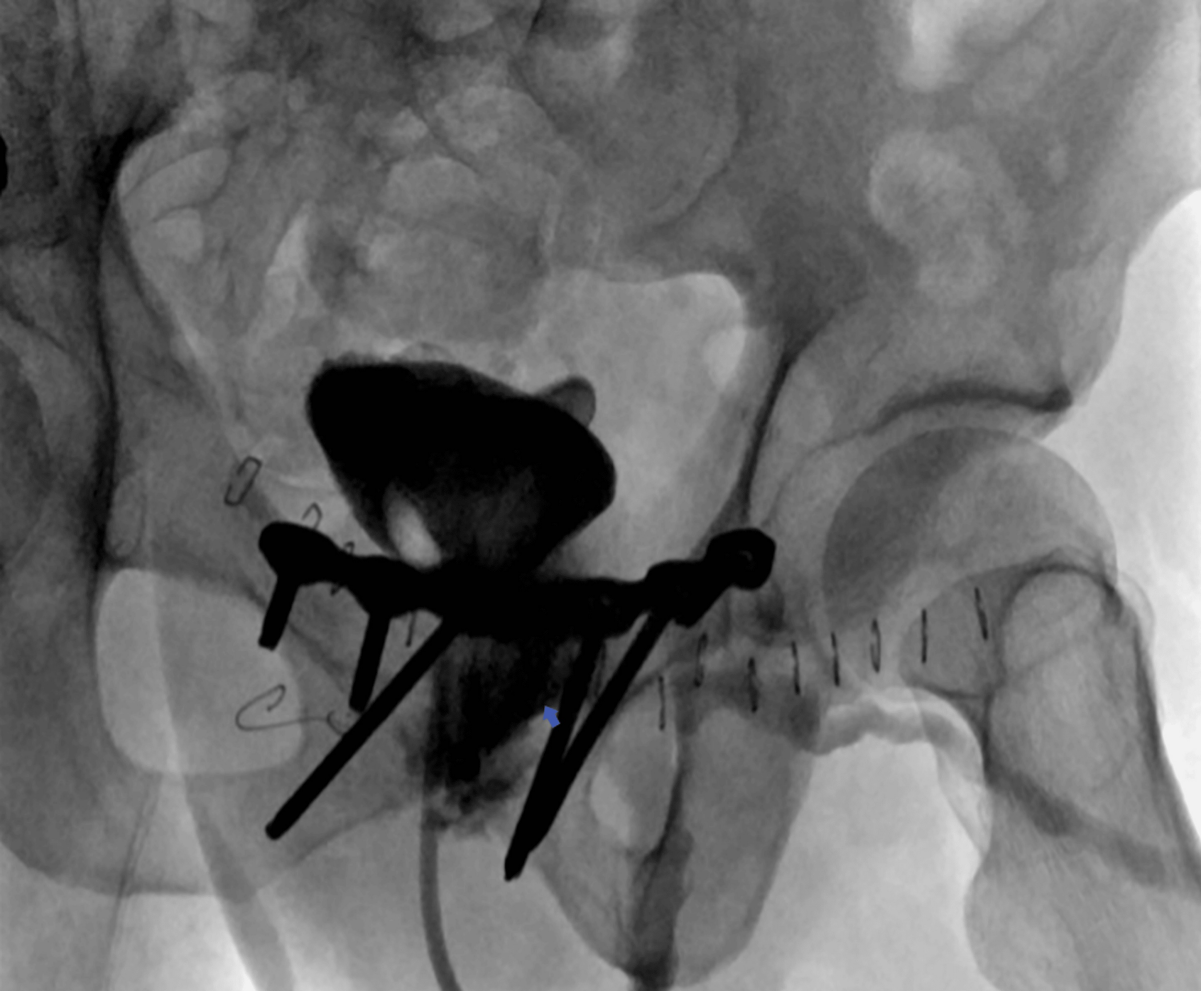
Retrograde cystography showing contrast extravasation (arrow) following bladder inflation, lateral view

Retrograde cystography was performed again (Figures 3, 4). It showed a 15 mm (transverse diameter) defect in the wall of the bladder anteriorly and inferiorly at 6 o’clock and an opacifying 7cm wide cavity anterior to the symphysis pubis extending to the left side. Anteriorly, it extends into the subcutaneous fat and opens into the skin.

**Figure 5:**
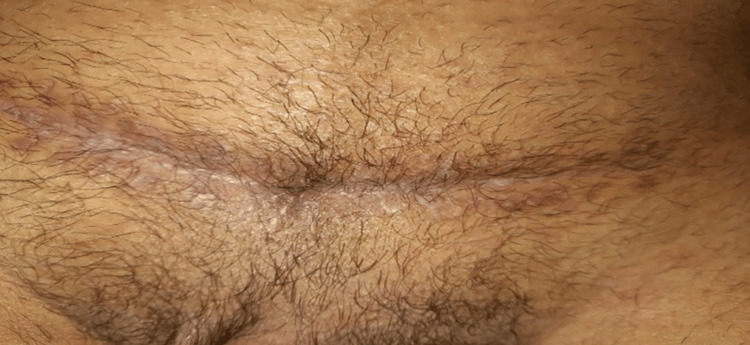
Wound appearance after three months; closure of the opening of the VCF can be seen

Purulent discharge from the Pfannenstiel incision along with urine raised suspicion about the presence of infected orthopedic instrumentation at the level of the pubic symphysis. A decision was made to attempt bone and wound debridement. During the procedure, a second attempt was made to close the bladder rupture using VICRYL 3-0 suture after the development of the retropubic space and identification of the bladder defect; this was followed by inflation of the bladder under pressure where no leak was visualized. The patient returned to the ward for monitoring and a Foley catheter was kept. In the following three days, the Pfannenstiel incision was clean with no pus or urine discharge. Then, gross hematuria developed for an unknown reason that led to clot retention and presumably bladder rupture, again. Urine leakage from the wound resumed thereafter. At this point, interventional radiology performed urine diversion with bilateral nephrostomy placement. Unfortunately, nephrostomies failed to completely divert urine away from the bladder and the urine leakage from the wound persisted, hence, bilateral obstructive ureteral balloons were placed along the nephrostomies to prevent urine from reaching the bladder. Unfortunately, spontaneous balloon displacement and deflation on one side prompted the removal of both balloons in the following 48 hours. At this point, with nephrostomy tubes on both sides, we decided to take a more conservative approach, that is, to watch and wait. Freeman et al. described a case closure of VCF that was successful using nephrostomy tubes and negative-pressure wound therapy. Therefore, in hopes of speeding up the VCF closure, we set up a mini-vacuum dressing over the wound using the UNO device (negative-pressure wound therapy adapted for smaller wounds) [3] while the bilateral nephrostomy tubes diverted urine away from the bladder [4]. With no further febrile illness or evidence of infection, the patient was discharged. At this point, he had bilateral nephrostomies, an indwelling Foley catheter, and the UNO device for vacuum dressing on the wound. Two weeks later, the fistula at the level of the wound closed with no further urine leakage (Figure 5). The UNO device was removed.

**Figure 6:**
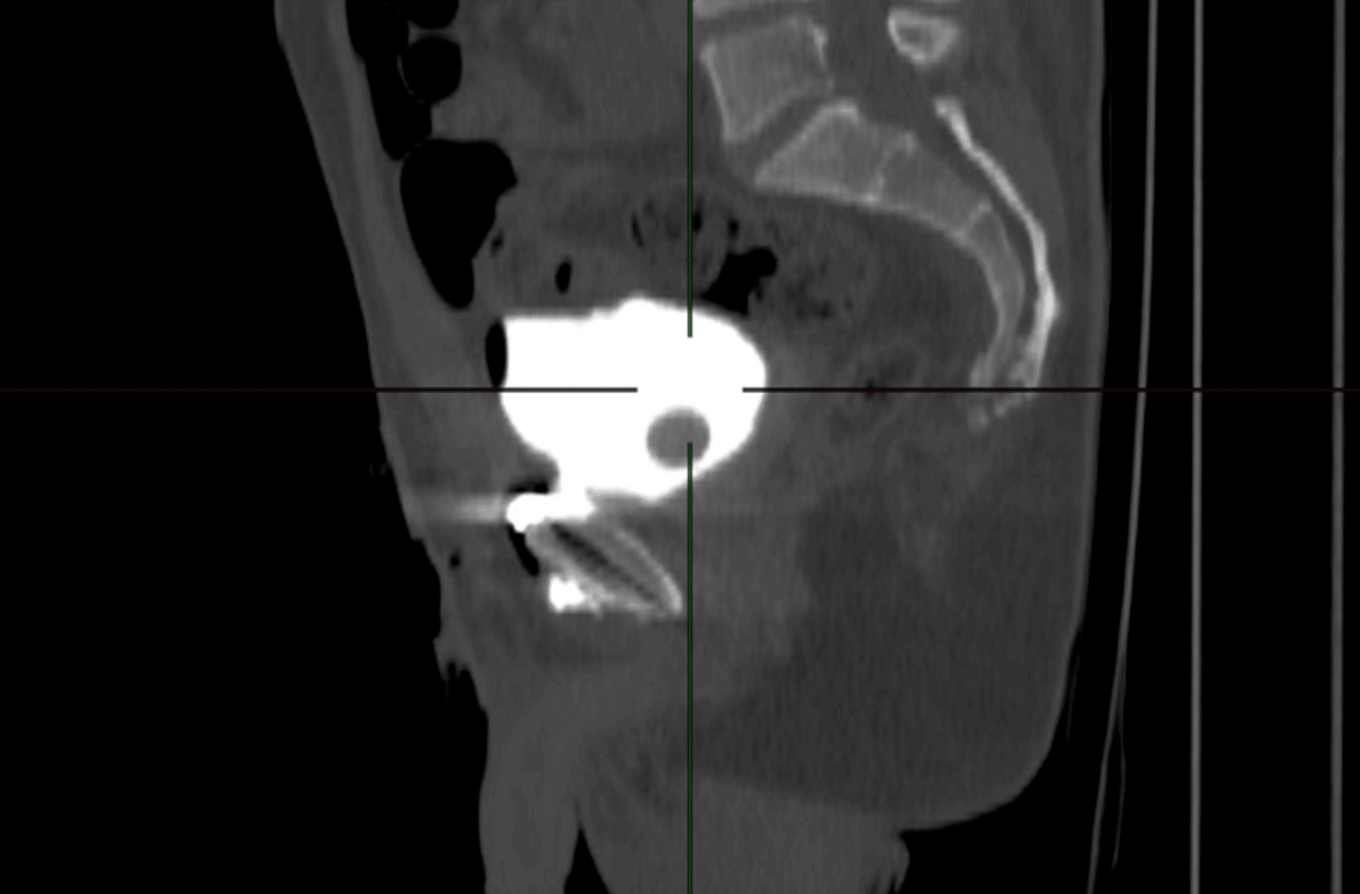
Retrograde CT cystography showing resolution of the VCF with no contrast extravasation

One month after discharge, a CT scan of the abdomen and pelvis showed complete resolution of the collection superior and anterior to the pubic symphysis with no evidence of bladder leakage after retrograde CT cystography (Figure 6). Bilateral nephrostomy tubes were removed followed by Foley catheter removal one week later, and the patient urinated normally thereafter.

In summary, the wound healed completely after 3 months and 16 days.

## Discussion

VCFs are abnormal tracts that arise between the bladder and the skin following surgery, trauma, radiation therapy, malignancy, etc [5-7]. Although they are a rare occurrence, they carry significant morbidity for the patient and are quite challenging to treat. Although surgical treatment may be the first option that comes to mind for a surgical or traumatic complication, our case underlines that it's not always feasible and/or efficient because of the distorted anatomy and often compromised tissues delaying healing and normal function restoration. When faced with this challenge, we had no choice but to give the tissue time to heal, hastening this process by the simultaneous application of novel techniques. Urinary diversion allowed for the temporary cessation of urine flow through the fistulous tract as suggested by Horenblas et al. [8], reducing local irritation and promoting healing. Negative pressure wound therapy (NPWT) played a crucial role in managing the wound and maintaining a clean and controlled environment conducive to tissue repair. Furthermore, the fistula itself can be complicated by infection [9], and adequate antibiotic administration is essential in the healing process and infection eradication.

This case underscores the importance of tailored, multidisciplinary approaches for managing complex fistulous conditions, particularly when surgery has failed. Here, conservative management can prove beneficial [10], with an emphasis on the importance of adjunctive therapies like NPWT in enhancing wound-healing dynamics. Future studies could further explore the long-term outcomes and refine standard treatment algorithms to help surgeons and clinicians improve the management of vesicocutaneous fistulas, ultimately ensuring a better quality of life for affected individuals.

## Conclusions

The treatment of vesicocutaneous fistulas (VCFs) can be challenging, as surgical therapy is not always successful. Minimally invasive techniques, such as urinary diversion and an indwelling urinary catheter to decompress the bladder, can be employed. Infection control is also essential to promote tissue healing. Refractory cases require a more insidious approach where time can prove beneficial and more novel techniques, such as vacuum therapy in our case, can be employed.
